# Possible detection of cervical spondylotic neuropathy using Distribution of F-latency (DFL), a new neurophysiological parameter

**DOI:** 10.1186/1756-0500-3-112

**Published:** 2010-04-23

**Authors:** Mohammad J Alam, Khondkar S Rabbani

**Affiliations:** 1 Department of Biomedical Physics & Technology, University of Dhaka, Dhaka, Bangladesh

## Abstract

**Background:**

We have previously reported a new nerve conduction measurement parameter which we named the Distribution of F-latency (DFL) and showed that this was an approximate mirror of the Distribution of Conduction Velocity (DCV) of motor nerve fibers. This work was performed using measurements on the 20 median nerves from 10 volunteers. The DFL showed a number of different patterns including single peaks, broad peaks and double peaks, the latter observed on subjects with Cervical Spondylosis (CS). It was thought that a retrospective analysis of these data could be worthwhile in determining whether Cervical Spondylotic neuropathy could be detected using the DFL.

**Findings:**

The DFL from the 8 median nerves of 4 normal subjects had single peaks, which has been assumed to represent a normal pattern. The DFL from one side of 5 subjects diagnosed with or suspected to have CS had double peaks. Broad peaks were observed in 7 nerves of which 5 were from subjects who had double peaks in the DFL on the contra lateral side.

**Conclusions:**

Based on these findings, double peaks in the DFL appear to be associated with CS neuropathy. These findings further suggest that broad peaks in the DFL could indicate the early stages of the disease. Differential compression of nerve branches at the spinal roots are being explored as possible causes. This study does not preclude other pathologies contributing to double or broad peaks, but does suggest that the DFL could form a screening tool for CS neuropathy.

## Background

We have previously reported a new nerve conduction parameter which we named the Distribution of F-latency (DFL) [1]. In this short report we propose a possible application of the DFL in screening for Cervical Spondylotic neuropathy, a common disorder of the aged population. This proposal originates from a retrospective analysis of the measurements used for establishing the DFL as a nerve conduction parameter, and relating the findings to the well known neurophysiological parameter - the Distribution of Conduction Velocity (DCV). From consideration of the basic physiological mechanism of F-responses and the statistical properties of random events contributing to the origin of the DFL, it was shown to approximately mirror the DCV of motor nerve fibers within a nerve trunk, particularly those that take part in the production of F-responses. Independent repeat samples of the DFL from 20 median nerves of 10 subjects showed a high average correlation (0.81 ± 0.15) between the corresponding repeated sets. While pursuing the above study we observed that subjects having diagnosed Cervical Spondylosis (CS) displayed a DFL pattern with double peaks which was very distinct from the single peak pattern observed in subjects having no neurological disease. DFL patterns with a broad peak, sometimes with indication of double humps, were also observed. These broad peaks were mostly seen in the median nerves from subjects where the contra lateral side showed a double peak in the DFL. These observations led us to investigate these results in more detail to see whether the changes in the DFL pattern were consistent with CS. If double peaks in the DFL indicate CS neuropathy, then a broad peak may indicate an early stage of the disease, and its detection could form a screening test which could be very helpful in terms of therapy and management.

## The study

DFL's were obtained from both median nerves of 10 adult subjects who volunteered for the test and gave informed consent. As part of our previous work on developing the DFL we demonstrated the very good repeatability of the measure by performing repeat measurements on the same nerve. Therefore the DFL patterns obtained were reproducible and representative of a particular nerve trunk. The subjects used in the development of the DFL were not chosen on any specific clinical criteria since the main objective of this earlier study was to determine the reproducibility of the DFL. Out of the 10 subjects studied, five did not report any neurological disorder, 4 had pain in the shoulder or numbness in the upper limbs, and had been clinically diagnosed with Cervical Spondylosis based on their symptoms and X-ray imaging. One of these subjects had been diagnosed with Cervical Spondylosis following an MRI examination. One subject, subject no. 6, complained of pain in both shoulders but had not consulted a doctor for clinical investigation of these symptoms prior to the study. However, the symptoms were consistent with CS. Since the objective of the study was to determine reproducibility, further clinical details were not deemed necessary.

**Figure 1:**
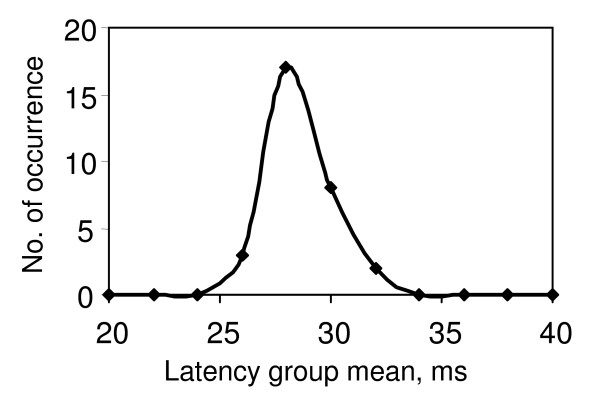
**Typical DFL with single peak, as obtained from normal subjects**.

**Figure 2:**
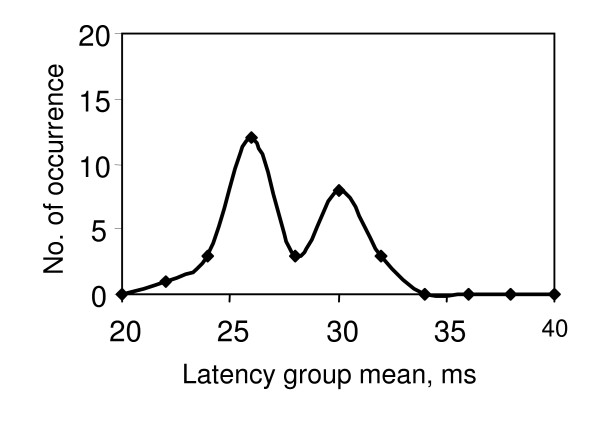
**Typical DFL with double peak, as obtained from subjects with CS**.

**Figure 3:**
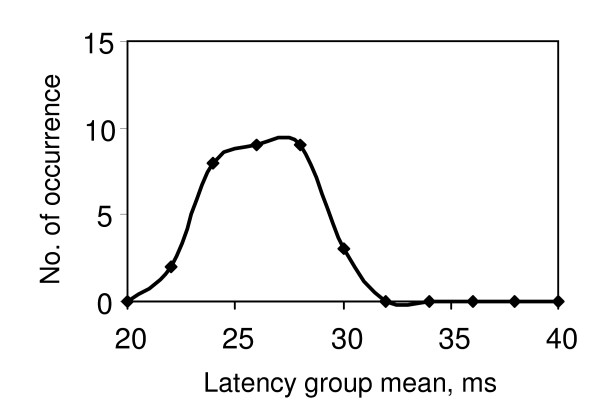
**Typical DFL with broad peak (plateau like, with two humps in some cases)**. Probably indicate early CS.

To obtain the DFL, 30 to 40 stimuli were applied to the median nerve at the wrist and the evoked EMG signals were obtained from the corresponding thenar muscle (abductor policies brevis). F-latencies for the resulting F-responses were sorted into latency 'bins' of width 2 ms. The number of occurrences in each bin was plotted against the latency mean and the resultant histogram smoothed to give the frequency distribution which is the DFL. Typically three patterns of DFL were observed as described earlier in this paper, (i) a single peak (S), ii) a clearly defined double peak (D), and (iii) a broad peak (B) - sometimes with the indication of two humps, and these are shown in Figs. 1, 2 &3 respectively. Each of the 20 DFL patterns obtained from the 10 subjects were verified through two independent data samples, thus giving 40 samples from the perspective of the present investigation.

The patterns of DFL were tabulated for the 20 median nerves (referred to as cases here) studied from the 10 subjects together with their clinical conditions to determine any relationship with CS. Table 1 presents these data on a subject basis whilst Table 2 presents these data in terms of the three observed patterns in the DFL.

**Table 1:** Correspondence of DFL patterns with neurological condition in the cervical region

**Subject No**.	Subject identifier	Age	Pattern of DFL*		Reported Clinical condition^#^	
		**Years**	**Right**	**Left**	**Right**	**Left**

1	MF	25	B	S	Nor	Nor

2	MJA	32	B	D	Nor	CS

3	MM	29	S	D	Nor	CS

4	OS	25	S	S	Nor	Nor

5	SI	23	B	S	Nor	Nor

6	SN	45	D	B	Pain	Pain

7	AS	40	D	B	CS	Nor

8	UK	28	D	B	CS	Nor

9	MR	26	S	B	Nor	Nor

10	RH	43	S	S	Nor	Nor

* S: Single peak; D: Clear double peak; B: Broad peak (with indication of double humps)

# Nor: Normal; CS: Clinically diagnosed Cervical Spondylosis; Pain: Pain in shoulder, not consulted a medical practitioner yet

**Table 2:** Collated summary of the correspondence of DFL patterns with neurological condition in the Cervical region

DFL pattern	No. of nerves	Clinical condition	Comments
Single Peak	8	All claimed normal	Single peak likely to represent normalcy

Double Peak	4	Diagnosed CS	Double peak likely to represent CS. Single case with pain possibly has CS

As above	1	Pain in shoulder, not yet gone for medical investigation	As above

Broad Peak (Plateau)	2	With single peak of DFL on opposite side	Cases with CS on one side are more likely to develop CS on the opposite side as well. Broad peak (plateau) may be an early indicator of CS.

As above	5	With double peak of DFL on opposite side, 4 with diagnosed CS, one with Pain in shoulder	As above

## Results

From tables 1 and 2 it can be seen that in all 8 cases having no neurological disorder, the DFL had single peaks.

Double peaks were observed in 5 cases. Four of these had diagnosed CS. The fifth case (subject no.6) probably had CS based on the symptoms reported.

A broad peak as shown in Fig.3 was observed in the DFL in 7 cases. Some of these DFLs had indications that two peaks were developing. Of these, 4 cases had diagnosed CS affecting the contra lateral side, and one (subject no. 6) had symptoms consistent with CS on the contra lateral side. The other 2 cases had no symptoms and had a single peak in the DFL on the contra lateral side.

From the data shown in Tables 1 and 2, it can be seen that the 8 cases that had a single peak in the DFL had no neurological symptoms. Therefore single peaks in the DFL appear to relate well to normal nerves. Since the DFL is an approximate mirror of the DCV for motor nerve fibers participating in the F-response, this would suggest that a single peak in the DCV would also represent the findings from a normal nerve. This is supported by earlier work on the DCV using collision techniques [2-4] and on the diameter distribution of large A-alpha myelinated fibres found from nerve biopsy [[5], p.64].

The four cases where CS was clinically diagnosed and the one case where symptoms were consistent with CS, all displayed double peaks in the DFL, a pattern that was clearly distinguishable from the single peaks found in normal subjects. The mechanism for the double peaks in CS is not currently clear, however, double peak in the DFL are not found in carpal tunnel syndrome (CTS), as we have previously reported [1]. CTS produced a simple delay in the whole DFL without a significant change in its shape. This finding may be anticipated from a physical consideration of nerve entrapment where a pressure acts across a nerve trunk. Pressure acting on all the nerve fibers equally will result in a time shift of the DFL pattern. Using the same argument, any entrapment enclosing the whole cross-section of the nerve trunk at or distal to the Brachial Plexus may be excluded. One possibility which is being explored by one of the authors (KSR) is that differential compression or degeneration of motor nerve branches near the spinal roots that innervate the thenar muscle could result in the double peaks seen in the DFL. Previous work shows that the thenar muscle is innervated by nerve branches from C8 and T1 [[6], p.859] and C7 [7]. Compression on one or more of these branches may contribute to a double, triple or a broad peak in the DFL depending on the degree of compression in each branch. This may suggest a causal relationship with radiculopathy caused by pressure on the cervical nerve branches, however, it is not currently possible to determine whether myelopathy caused by a pressure on the spinal cord itself may lead to modification of the DFL pattern.

As observed, seven cases displayed a broad peak in the DFL, with a well defined plateau region and some indication of the onset of two peaks as shown in Fig.3. Table 2 shows that 5 of these cases had a double peak in the DFL on the contra lateral side where there was either diagnosed CS (4 cases) or symptoms consistent with CS (subject 6). If someone has CS affecting one side, there is a high probability that the other side may become affected in the future. Therefore, such broad peaks may indicate the early stages of CS, which has yet to produce clinical symptoms. This leads us to speculate that the 2 cases with broad peak on one side and single peak on the other may indicate early stage CS on the side with the broad peak. In neither case were there clinical symptoms. However, this possibility can only be substantiated through MRI investigations.

Conventional neurophysiological techniques have so far not produced a method to give a positive diagnosis of CS other than by exception where they exclude other peripheral nerve disorders. Using a train of 200 stimuli, Peioglou-Harmaoussi et al [8] studied the frequency of occurrence of F-responses and wave shapes from the ulnar nerve and their relationship to CS. Neither of these provided a method to identify CS as differences with CS were not well defined. However, there appears to be consistency between one of their observations and our observations for the DFL. They found that the F-wave appeared visually complex in CS subjects when compared with those from controls. In our findings, the DFL is a simple distribution with a single peak for normal subjects, therefore the population of nerve cells has less dispersion in their latency which in turn would produce simple F-waves from the summed individual motor unit responses. Conversely, in CS, the DFL has a greater dispersion with double peaks, suggesting that a greater spread of latencies which would lead to more complex patterns in the F waves.

## Conclusions

Findings from the present work, together with the initial hypotheses on the creation of double peaks in the DFL suggests that that CS has a very likely association with double peaks in the DFL when compared with the single peak found in normal subjects. If this is correct, then a broad peak in the DFL may represent the early stages of CS. However, further studies are necessary to determine whether any other neuropathy contributes to such double peaks in the DFL. The present study also suggests that the DFL has the potential to be used as a screening tool for CS neuropathy, particularly in the early stages of the disease before clinical symptoms occur. The findings presented in this paper justifies further work on the DFL, specifically in correlating the findings from MRI scans of nerves and the DFL to investigate the underlying physiological mechanisms and to further explore the associations between patterns in the DFL and CS.

We have made DFL a routine investigation at the clinical centre where this study was carried out. Double, (and sometimes triple) peaks in the DFL were observed from the thenar muscle in many patients presenting with symptoms matching those of CS neuropathy. Double peaks in the DFL were also observed for the tibial and common peroneal nerves of some patients indicating a lumbo-sacral (LS) spondylotic neuropathy. Therefore further investigation into the use of DFL for both CS and LS neuropathy may lead to a new and simple diagnostic tool.

The only reliable diagnostic tool for CS at present is MRI which is expensive and not widely available in Third world countries. Plain film X-ray cannot image a lesion in the nerve branches or in the spinal cord unless a contrast agent is used which is rather traumatic for the patient. An electrophysiological technique like DFL can be made available widely with much reduced equipment cost when compared with imaging techniques, and with almost zero running cost. Therefore, if DFL is successful in detecting CS unambiguously, it may become an important neuro-diagnostic tool. Particularly if the broad plateau-like peak can be established to be an early indicator of CS, this will have the potential to contribute to the treatment and management of CS. Since CS is a common disorder afflicting most people above the age of fifty, such a simple and straightforward diagnostic technique may have profound implications in healthcare, improving diagnosis whilst reducing costs.

## Competing interests

The authors declare that they have no competing interests.

## Authors' contributions

MJA performed the experimental measurements, made the necessary modifications to the software to plot the DFLs from raw data, and first noted the difference in DFL patterns between subjects with CS and normal subjects. This work was carried out during his M.Phil research. KSR conceived of the basic concepts of the DFL and designed the experiments to verify its relationship to DCV. He also carried out the tabular analysis of the DFL and available clinical data in the present work, presented the arguments, and wrote the whole paper. He supervised MJA for his M.Phil degree. Both authors read and approved the final manuscript.

## Authors' information

MJA carried out this research as a part of his M.Phil degree requirements, under the supervision of KSR at Dhaka University, Bangladesh. He has Bachelor's and Master's degrees in Physics and subsequently assisted KSR in routine clinical work on nerve conduction on patients.

KSR got his Bachelor's degree in Physics from Dhaka University, Bangladesh, Master's degrees in Physics from Islamabad University, Pakistan, and Ph.D. in Microelectronics from Southampton University, UK. Returning home he joined the Physics department of Dhaka University in a teaching position and developed an interest in Medical Physics soon after. Through a ten year academic link with the department of Medical Physics & Clinical Engineering, Sheffield University, UK (1983-1992) he experienced different areas of this discipline and strived to develop his own capability and expertise. One of these areas was nerve conduction and with the University of Sheffield's help he made the hardware and software for the computerized equipment that has being used in Bangladesh since 1988 for routine clinical investigations; he himself giving the clinical reports. This has given KSR expertise in both the physics and clinical aspects of nerve conduction, together with the instrumentation.
